# Methyl­ergometrine maleate from synchrotron powder diffraction data

**DOI:** 10.1107/S1600536809050351

**Published:** 2009-11-28

**Authors:** Jan Rohlíček, Michal Hušák, Bohumil Kratochvíl, Alexandr Jegorov

**Affiliations:** a Institute of Chemical Technology Prague, Technická 5, 16628 Prague 6, Czech Republic; b Teva Czech Industries s.r.o., R&D, Branišovská 31, 370 05 České Budějovice, Czech Republic

## Abstract

The title compound {systematic name: 9,10-didehydro-*N*-[1-(hydroxy­meth­yl)prop­yl]-d-lysergamide maleate}, C_20_H_26_N_3_O_2_^+^·C_4_H_3_O_4_^−^, contains a large rigid ergolene group. This group consists of an indole plane connected to a six-membered carbon ring adopting an envelope conformation and *N*-methyl­tetra­hydro­pyridine where the methyl group is in an equatorial position. In the crystal, inter­molecular N—H⋯O, O—H⋯N and O—H⋯O hydrogen bonds form an extensive three-dimensional hydrogen-bonding network, which holds the cations and anions together.

## Related literature

For background to ergometrine, see: Dudley & Moir (1935 ▶); Kharasch & Legault (1935 ▶). Formethyl­ergometrine, see Stoll & Hofmann (1943 ▶). For crystal structure determinations of ergometrine, see: Čejka *et al.* (1996 ▶); Hušák *et al.* (1998 ▶).
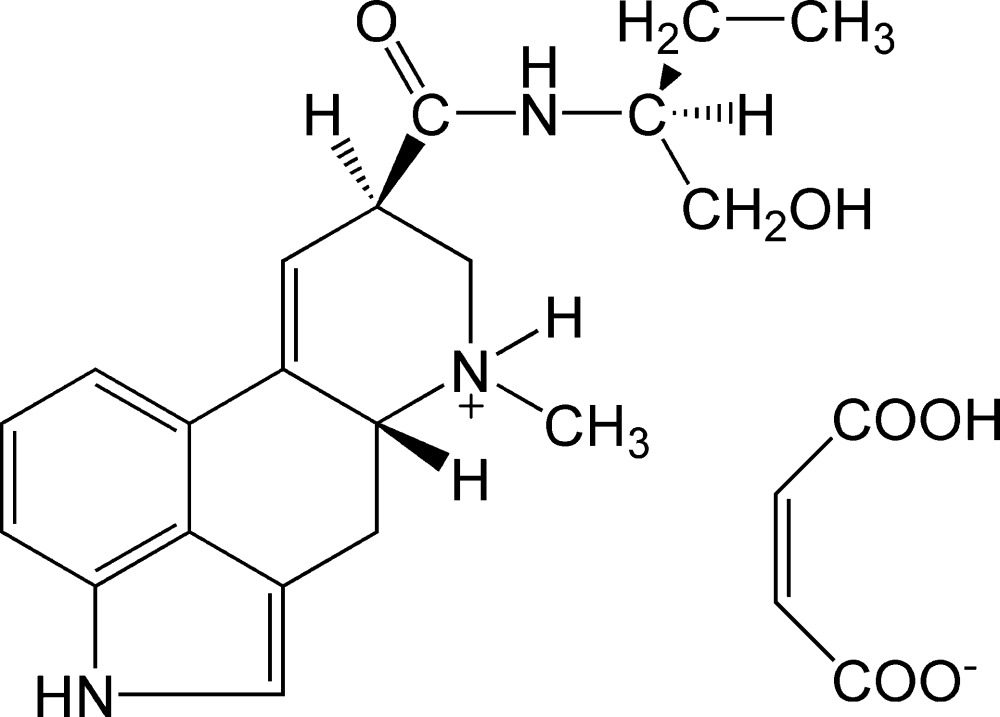


## Experimental

### 

#### Crystal data

C_20_H_26_N_3_O_2_^+^·C_4_H_3_O_4_^−^
*M* *_r_* = 455.51
Orthorhombic, 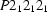

*a* = 5.71027 (5) Å
*b* = 12.76978 (17) Å
*c* = 33.1455 (4) Å
*V* = 2416.93 (5) Å^3^
*Z* = 4
Synchrotron radiation
λ = 0.6996 Å
*T* = 293 K
Specimen shape: cylinder
40 × 1 × 1 mm
Specimen prepared at 101 kPa
Specimen prepared at 293 K
Particle morphology: needle, white

#### Data collection

BM01B, ESRF, Grenoble
Specimen mounting: 1.0 mm borosilicate glass capillary
Specimen mounted in transmission mode
Scan method: step
Absorption correction: none
2θ_min_ = 0.5, 2θ_max_ = 29.5°
Increment in 2θ = 0.003°

#### Refinement

*R*_p_ = 0.060
*R*_wp_ = 0.080
*R*_exp_ = 0.021
*R*_B_ = 0.088
*S* = 3.76
Wavelength of incident radiation: 0.6996 Å
Excluded region(s): none
Profile function: pseudo-Voigt profile coefficients as parameterized in Thompson *et al.* (1987 ▶), asymmetry correction according to Finger *et al.* (1994 ▶)
617 reflections
100 parameters
96 restraints
H-atom parameters not refined
Preferred orientation correction: March–Dollase (Dollase, 1986 ▶); direction of preferred orientation - 011, MD = 1.26

### 

Data collection: ESRF *SPEC* package (Certified Scientific Software, 2003 ▶); cell refinement: *GSAS* (Larson & Von Dreele, 1994 ▶); data reduction: *CRYSFIRE* (Shirley, 2000 ▶); program(s) used to solve structure: *FOX* (Favre-Nicolin & Černý, 2002 ▶); program(s) used to refine structure: *GSAS*; molecular graphics: *Mercury* (Macrae *et al.*, 2006 ▶) and *PLATON* (Spek, 2003 ▶); software used to prepare material for publication: *enCIFer* (Allen *et al.*, 2004 ▶).

## Supplementary Material

Crystal structure: contains datablocks global, I. DOI: 10.1107/S1600536809050351/cv2630sup1.cif

Rietveld powder data: contains datablocks I. DOI: 10.1107/S1600536809050351/cv2630Isup2.rtv

Additional supplementary materials:  crystallographic information; 3D view; checkCIF report

## Figures and Tables

**Table 1:** Hydrogen-bond geometry (Å, °)

*D*—H⋯*A*	*D*—H	H⋯*A*	*D*⋯*A*	*D*—H⋯*A*
O2*s*—H202⋯O4*s*	1.20	1.28	2.479 (5)	179
N13—H131⋯O3*s*	0.86	1.77	2.634 (4)	173
O23—H232⋯O19^i^	0.83	2.12	2.925 (8)	160
N20—H201⋯O1*s* ^ii^	0.87	2.04	2.912 (5)	177
N1—H11⋯O19^iii^	0.88	2.03	2.852 (4)	154

Symmetry codes: (i) 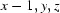; (ii) 
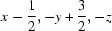
; (iii) 
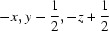
.

**Fig. 1.**
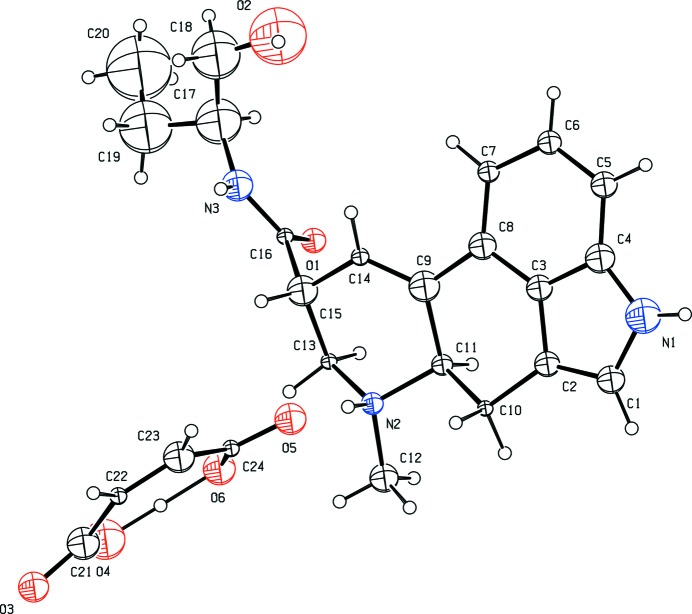
The molecular structure of methylergometrine maleate showing the atomic numbering. Displacement spheres are drawn at the 30% probability level.

**Fig. 2.**
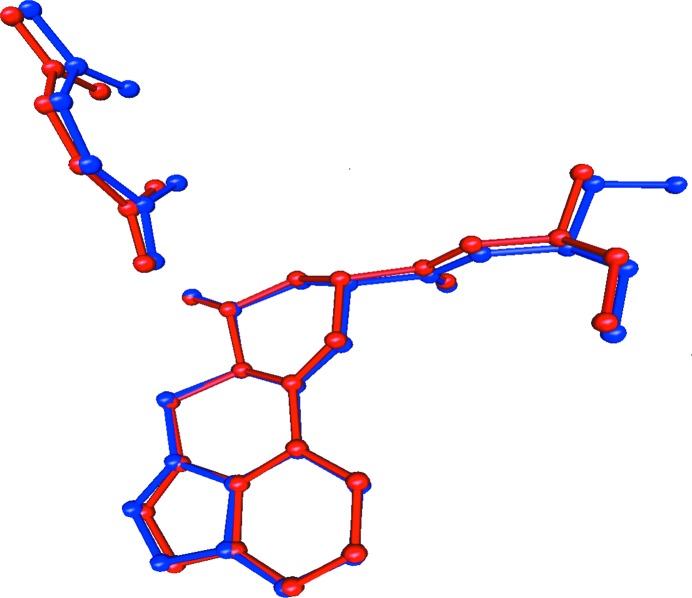
Overlaid asymmetric parts of unit cells of methylergometrine maleate (blue) and ergometrine maleate (red)

**Fig. 3.**
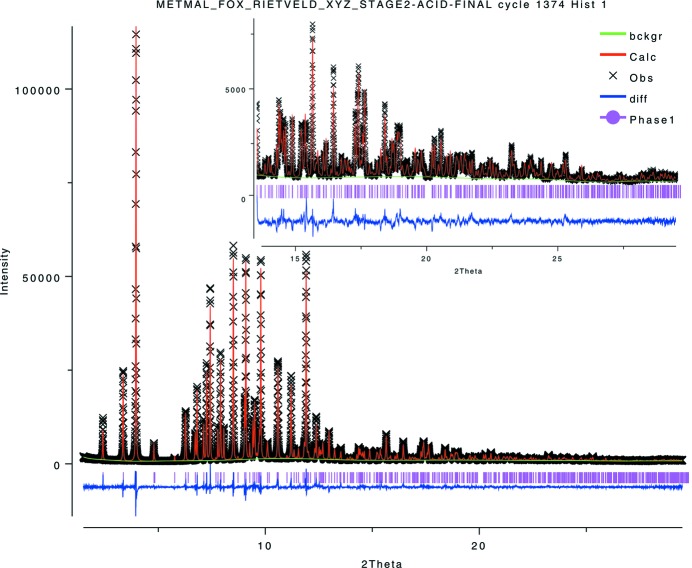
The final Rietveld plot showing the measured data (black thin-plus), calculated data (red line) and difference curve (blue line). Calculated positions of the reflection are shown by vertical bars.

## supplementary crystallographic information

### Comment

Methylergometrine is a semisynthetic ergot alkaloid derived from (+)-lysergic acid and (*S*)-(+)-2-amino-1-butanol (Stoll & Hofmann, 1943). It is nearly isostructural with natural ergot alkaloid ergometrine maleate (Čejka *et al.*, 1996). Previous attempts to solve this structure by molecular modeling using ergometrine maleate as the starting model were successful, but the result was not very precise (Čejka *et al.*, 1996). Hence the crystal structure was not published. In this paper we report crystal structure determination of the title compound (I) from synchrotron powder diffraction data.

The asymmetric unit of (I) contains a methylergometrinium cation and one molecule of maleate (Fig. 1). All bond lengths and angles in (I) are comparable with reported structure of ergometrine maleate (Čejka *et al.*, 1996). The molecule of maleate is situated in the same position and the hydrogen bonding system is practically the same. Intermolecular N—H···O, O—H···N and O—H···O hydrogen bonds (Table 1) form an extensive three-dimensional hydrogen-bonding network which held cations and anions together.

### Experimental

Crystallization of methylergometrine maleate from various solvents (alcohols, acetic acid esters, acetone, dioxane, dimethyl sulphoxide) provided hair-like long needle crystals in all cases. One crystalline form with distinct powder patterns was found.

### Refinement

The powder diffraction data measurement was done on BM01B beamline (Swiss-Norwegian Beamlines) at the ESRF, Grenoble. Before the measurement the diffractometer was calibrated by using LaB_6 _standard sample and the value of wavelength was checked (0.6996 Å). The powder sample was placed in a 1 mm capillary. The measurement was done at room temperature. The capillary was rotating during the data collection. The diffractogram was measured from 0.515° to 29.49° 2θ with 0.0025° step scan and the sample was irradiated for 1 s per step. The data from all six detectors were finally binned.

The indexation confirmed unit-cell parameters and space group obtained from previous measurement (Čejka *et al.*, 1996): *a *=5.71 Å, *b*=12.77 Å, *c* =33.15 Å, *Z* = 4, V = 2 417 Å^3^, *P2_1_2_1_2_1_*. Molecule of ergometrine (Čejka *et al.*, 1996) was used as a starting model for structure solution. This model was transferred to the *z*-matrix and the missing methyl group was added in the standard C—C distance (1.52 Å). This way changed *z*-matrix was loaded into the program FOX (Favre-Nicolin & Černý, 2002) and structure was solved by using parallel tempering algorithm. The structure solution result confirmed similarity with ergometrine maleate, see Fig.2. Refinement of this result was carried out in *GSAS* (Larson & Von Dreele, 1994). Hydrogen atoms were placed in their theoretical positions and structure was refined with bonds, angles and planar groups restraints (N1—C10,C9/C10/C12/C16, C17/C18/O19/N20, C6s/C5s/O1s/O2s, C7s/C8s/O3s/O4s andC5s/C6s/C7s/C8s). All atomic coordinates and *U*_iso_ parameters of non-hydrogen atoms were refined. Hydrogen atoms were not refined, it was necessary to relocate H atoms into the correct positions after few cycles. Hydrogen atom H202 was manually placed between oxygen atoms O2s and O4s. At the final stage of the refinement, only atomic coordinates of non-hydrogen atoms were refined to the final agreement factors *R_p_* = 0.0631 and *R_wp_* = 0.0831. The diffraction profiles and differences between the measured and calculated profiles are shown in Fig. 3.

### Crystal data

**Table:**

C_20_H_26_N_3_O_2_^+^·C_4_H_3_O_4_^−^	*F*(000) = 960.0
*M**_r_* = 455.51	*D*_x_ = 1.246 Mg m^−^^3^
Orthorhombic, *P*2_1_2_1_2_1_	Synchrotron radiation, λ = 0.6996 Å
*a* = 5.71027 (5) Å	*T* = 293 K
*b* = 12.76978 (17) Å	Particle morphology: needle
*c* = 33.1455 (4) Å	white
*V* = 2416.93 (5) Å^3^	cylinder, 40 × 1 mm
*Z* = 4	Specimen preparation: Prepared at 293 K and 101 kPa

### Data collection

**Table:**

ID31 diffractometer	Data collection mode: transmission
Radiation source: X-Ray	Scan method: step
Si(111)	2θ_min_ = 0.52°, 2θ_max_ = 29.49°, 2θ_step_ = 0.003°
Specimen mounting: 1.0 mm borosilicate glass capillary	

### Refinement

**Table:**

Least-squares matrix: full	Profile function: Pseudo-Voigt profile coefficients as parameterized in Thompson *et al.* (1987), asymmetry correction according to Finger *et al.* (1994)
*R*_p_ = 0.060	100 parameters
*R*_wp_ = 0.080	96 restraints
*R*_exp_ = 0.021	0 constraints
*R*_Bragg_ = 0.088	H-atom parameters not refined
*R*(*F*^2^) = 0.08232	Weighting scheme based on measured s.u.'s *w* = 1/σ(Y_obs_)^2^
χ^2^ = 14.138	(Δ/σ)_max_ = 0.03
11591 data points	Background function: Shifted Chebyschev
Excluded region(s): no	Preferred orientation correction: March–Dollase (Dollase, 1986); direction of preferred orientation - 011, MD = 1.26

### Fractional atomic coordinates and isotropic or equivalent isotropic displacement parameters (Å^2^)

**Table:**

	*x*	*y*	*z*	*U*_iso_*/*U*_eq_	
N1	−0.2972 (5)	0.6888 (3)	0.29856 (7)	0.07305*	
C2	−0.1311 (5)	0.6344 (2)	0.27627 (8)	0.04511*	
C3	−0.0706 (4)	0.6910 (2)	0.24331 (7)	0.03699*	
C4	−0.2019 (3)	0.7843 (2)	0.24509 (6)	0.03942*	
C5	−0.3442 (4)	0.7819 (3)	0.27994 (6)	0.05116*	
C6	−0.4884 (4)	0.8647 (3)	0.28782 (7)	0.04053*	
C7	−0.4901 (4)	0.9470 (3)	0.26187 (8)	0.03292*	
C8	−0.3485 (4)	0.9500 (2)	0.22692 (7)	0.02676*	
C9	−0.2006 (3)	0.86750 (18)	0.21789 (6)	0.04409*	
C10	−0.0383 (3)	0.85683 (16)	0.18355 (6)	0.05585*	
C11	0.0703 (5)	0.66917 (18)	0.20642 (8)	0.01406*	
C12	0.1520 (3)	0.77235 (18)	0.18765 (6)	0.02621*	
N13	0.2572 (4)	0.7525 (2)	0.14658 (7)	0.02834*	
C14	0.4543 (6)	0.6780 (3)	0.14769 (10)	0.04728*	
C15	0.3333 (4)	0.8518 (2)	0.12743 (7)	0.0149*	
C16	−0.0506 (3)	0.9178 (3)	0.15108 (8)	0.01827*	
C17	0.1276 (3)	0.9217 (2)	0.11776 (6)	0.05457*	
C18	0.2054 (3)	1.0353 (2)	0.11406 (5)	0.01555*	
O19	0.3842 (5)	1.0675 (3)	0.13078 (10)	0.03564*	
N20	0.0695 (5)	1.0956 (2)	0.09146 (10)	0.0567*	
C21	0.0846 (6)	1.2096 (2)	0.08735 (7)	0.12048*	
C22	−0.1555 (8)	1.2575 (4)	0.09008 (13)	0.13815*	
O23	−0.2828 (7)	1.2409 (7)	0.12481 (15)	0.18992*	
C24	0.1859 (9)	1.2439 (4)	0.04775 (12)	0.16384*	
C25	0.254 (2)	1.3598 (5)	0.0502 (2)	0.24862*	
O1s	0.1598 (8)	0.5073 (3)	−0.05735 (10)	0.05346*	
O2s	0.3200 (5)	0.6095 (3)	−0.01229 (11)	0.09409*	
O3s	−0.0518 (7)	0.6674 (3)	0.09751 (9)	0.05776*	
O4s	0.2452 (6)	0.6788 (3)	0.05636 (10)	0.06078*	
C5s	0.1379 (6)	0.56141 (17)	−0.02617 (8)	0.06152*	
C6s	−0.0894 (5)	0.57248 (19)	−0.00445 (9)	0.0169*	
C7s	−0.1334 (5)	0.61043 (19)	0.03209 (9)	0.05644*	
C8s	0.0294 (5)	0.65414 (14)	0.06283 (8)	0.01622*	
H21	−0.0702	0.5649	0.2836	0.0541*	
H61	−0.5833	0.8631	0.3116	0.0486*	
H71	−0.5868	1.0027	0.268	0.0395*	
H81	−0.3514	1.0068	0.2101	0.0324*	
H111	0.2034	0.6252	0.2141	0.0169*	
H112	−0.0222	0.629	0.1881	0.0169*	
H121	0.273	0.7969	0.2049	0.0315*	
H141	0.5132	0.6649	0.1216	0.0567*	
H142	0.5746	0.7014	0.165	0.0567*	
H143	0.4012	0.6094	0.1585	0.0567*	
H151	0.4358	0.8844	0.1458	0.0179*	
H152	0.4147	0.8326	0.1036	0.0179*	
H161	−0.1831	0.9588	0.1491	0.0219*	
H171	0.0579	0.8964	0.0936	0.0655*	
H211	0.1864	1.2337	0.1082	0.1804*	
H221	−0.1257	1.3314	0.0901	0.2258*	
H222	−0.2393	1.2362	0.0685	0.2258*	
H241	0.0678	1.2255	0.0272	0.1966*	
H242	0.3175	1.1955	0.0415	0.1966*	
H251	0.3132	1.3721	0.0222	0.4183*	
H252	0.118	1.3943	0.0536	0.4183*	
H253	0.3677	1.3643	0.068	0.4183*	
H601	−0.223	0.549	−0.019	0.0203*	
H701	−0.292	0.61	0.04	0.0677*	
H11	−0.3603	0.6651	0.3211	0.0877*	
H201	−0.0502	1.0631	0.0811	0.068*	
H131	0.148	0.725	0.132	0.0411*	
H202	0.285	0.643	0.021	0.1129*	
H232	−0.3996	1.2015	0.1235	0.27*	

### Geometric parameters (Å, °)

**Table:**

N13—C14	1.474 (4)	N13—H131	0.86
N13—C15	1.483 (4)	N20—H201	0.87
N20—C18	1.325 (4)	C2—H21	0.98
N20—C21	1.465 (4)	C6—H61	0.96
C2—C3	1.355 (4)	C7—H71	0.92
C3—C4	1.409 (3)	C8—H81	0.91
C3—C11	1.490 (4)	C11—H111	0.98
C4—C5	1.413 (3)	C11—H112	0.95
C4—C9	1.393 (3)	C12—H121	0.95
C5—C6	1.365 (5)	C14—H141	0.94
C6—C7	1.358 (5)	C14—H142	0.94
C7—C8	1.413 (3)	C14—H143	0.99
C8—C9	1.383 (3)	C15—H151	0.94
C9—C10	1.474 (3)	C15—H152	0.95
C10—C12	1.538 (3)	C16—H161	0.92
C10—C16	1.330 (4)	C17—H171	0.95
C11—C12	1.530 (3)	C21—H211	0.95
C15—C17	1.510 (3)	C22—H221	0.96
C16—C17	1.503 (3)	C22—H222	0.90
C17—C18	1.522 (4)	C24—H241	0.99
C21—C22	1.504 (6)	C24—H242	0.99
C21—C24	1.500 (5)	C25—H251	1.00
C24—C25	1.532 (9)	C25—H252	0.90
O23—H232	0.84	C25—H253	0.88
N1—H11	0.88		
			
C2—N1—C5	109.2 (2)	C3—C2—H21	126
C12—N13—C14	112.9 (2)	C5—C6—H61	119
C12—N13—C15	111.0 (2)	C7—C6—H61	122
C14—N13—C15	109.8 (2)	C6—C7—H71	117
C18—N20—C21	126.6 (3)	C8—C7—H71	120
N1—C2—C3	109.7 (2)	C7—C8—H81	121
C2—C3—C4	106.4 (2)	C9—C8—H81	119
C2—C3—C11	134.4 (2)	C3—C11—H111	108
C4—C3—C11	118.7 (2)	C3—C11—H112	109
C3—C4—C5	108.8 (2)	C12—C11—H111	111
C3—C4—C9	127.96 (19)	C12—C11—H112	112
C5—C4—C9	123.3 (2)	H111—C11—H112	107
N1—C5—C4	106.0 (3)	N13—C12—H121	108
N1—C5—C6	134.9 (2)	C10—C12—H121	110
C4—C5—C6	119.1 (3)	C11—C12—H121	105
C5—C6—C7	118.8 (2)	N13—C14—H141	111
C6—C7—C8	122.4 (3)	N13—C14—H142	112
C7—C8—C9	120.4 (2)	N13—C14—H143	110
C4—C9—C8	115.97 (19)	H141—C14—H142	111
C4—C9—C10	115.61 (18)	H141—C14—H143	106
C8—C9—C10	128.4 (2)	H142—C14—H143	106
C9—C10—C12	116.18 (17)	N13—C15—H151	106
C9—C10—C16	122.53 (19)	N13—C15—H152	106
C12—C10—C16	121.28 (18)	C17—C15—H151	111
C3—C11—C12	109.71 (19)	C17—C15—H152	111
N13—C12—C10	108.63 (17)	H151—C15—H152	110
N13—C12—C11	110.09 (19)	C10—C16—H161	116
C10—C12—C11	115.12 (17)	C17—C16—H161	119
N13—C15—C17	111.62 (19)	C15—C17—H171	108
C10—C16—C17	125.4 (2)	C16—C17—H171	109
C15—C17—C16	110.6 (2)	C18—C17—H171	112
C15—C17—C18	110.70 (16)	N20—C21—H211	107
C16—C17—C18	106.8 (2)	C22—C21—H211	112
O19—C18—N20	123.1 (3)	C24—C21—H211	108
O19—C18—C17	121.6 (2)	O23—C22—H221	104
N20—C18—C17	115.37 (19)	O23—C22—H222	110
N20—C21—C22	110.2 (3)	C21—C22—H221	104
N20—C21—C24	113.2 (3)	C21—C22—H222	108
C22—C21—C24	106.6 (3)	H221—C22—H222	113
O23—C22—C21	117.9 (4)	C21—C24—H241	106
C21—C24—C25	109.5 (4)	C21—C24—H242	107
C22—O23—H232	118	C25—C24—H241	116
C2—N1—H11	124	C25—C24—H242	115
C5—N1—H11	127	H241—C24—H242	103
C12—N13—H131	107	C24—C25—H251	101
C14—N13—H131	108	C24—C25—H252	105
C15—N13—H131	109	C24—C25—H253	107
C18—N20—H201	114	H251—C25—H252	109
C21—N20—H201	119	H251—C25—H253	111
N1—C2—H21	124	H252—C25—H253	121

### Hydrogen-bond geometry (Å, °)

**Table:**

*D*—H···*A*	*D*—H	H···*A*	*D*···*A*	*D*—H···*A*
O2s—H202···O4s	1.20	1.28	2.479 (5)	179
N13—H131···O3s	0.86	1.77	2.634 (4)	173
O23—H232···O19^i^	0.83	2.12	2.925 (8)	160
N20—H201···O1s^ii^	0.87	2.04	2.912 (5)	177
N1—H11···O19^iii^	0.88	2.03	2.852 (4)	154

Symmetry codes: (i) *x*−1, *y*, *z*; (ii) *x*−1/2, −*y*+3/2, −*z*; (iii) −*x*, *y*−1/2, −*z*+1/2.

## References

[bb1] Allen, F. H., Johnson, O., Shields, G. P., Smith, B. R. & Towler, M. (2004). *J. Appl. Cryst.* **37**, 335–338.

[bb2] Čejka, J., Hušák, M., Kratochvíl, B., Jegorov, A. & Cvak, L. (1996). *Coll. Czech. Chem. Commun.* **61** 1396–1404.

[bb3] Certified Scientific Software (2003). *SPEC*. Certified Scientific Software, Cambridge, MA, USA.

[bb4] Dollase, W. A. (1986). *J. Appl. Cryst.* **19**, 267–272.

[bb5] Dudley, H. W. & Moir, C. (1935). *Br. Med. J* **1**, 520–523. 10.1136/bmj.1.3871.520 PMC2459740 20778930

[bb6] Favre-Nicolin, V. & Černý, R. (2002). *J. Appl. Cryst.* **35**, 734–743.

[bb7] Finger, L. W., Cox, D. E. & Jephcoat, A. P. (1994). *J. Appl. Cryst.* **27**, 892–900.

[bb8] Hušák, M., Kratochvíl, B. & Jegorov, A. (1998). *Z. Kristallogr.* **213**, 195–196.

[bb9] Kharasch, M. S. & Legault, R. R. (1935). *Science*, **81**, 388. 10.1126/science.81.2103.388 17769437

[bb10] Larson, A. C. & Von Dreele, R. B. (1994). *GSAS*. Report LAUR 86-748. Los Alamos National Laboratory, New Mexico, USA.

[bb11] Macrae, C. F., Edgington, P. R., McCabe, P., Pidcock, E., Shields, G. P., Taylor, R., Towler, M. & van de Streek, J. (2006). *J. Appl. Cryst.* **39**, 453–457.

[bb12] Shirley, R. (2000). *CRYSFIRE User’s Manual*. Guildford, England: The Lattice Press.

[bb13] Spek, A. L. (2003). *J. Appl. Cryst.* **36**, 7–13.

[bb14] Stoll, A. & Hofmann, A. (1943). *Helv. Chim. Acta*, **26**, 944–965.

[bb15] Thompson, P., Cox, D. E. & Hastings, J. B. (1987). *J. Appl. Cryst.* **20**, 79–83.

